# Possible Signals of Ocular Disorders Associated with Gabapentinoids: A Three-Arm Study

**DOI:** 10.3390/ph19081175

**Published:** 2026-07-27

**Authors:** Mya Murray, Amira Guirguis, Paul Deslandes, John Martin Corkery, Stefania Chiappini, Mariacristina Parravano, Fabrizio Schifano

**Affiliations:** 1 Pharmacy Department, Swansea University, Swansea SA2 8PP, UK; 2112728@swansea.ac.uk (M.M.); p.n.deslandes@swansea.ac.uk (P.D.); 2 Psychopharmacology, Drug Misuse, and Novel Psychoactive Substances Research Unit, University of Hertfordshire, Hatfield AL10 9AB, UK; j.corkery@herts.ac.uk (J.M.C.);; 3 Faculty of Medicine and Surgery, UniCamillus International Medical University, Via di S. Alessandro 8, 00131 Rome, Italy; stefaniachiappini9@gmail.com; 4 Department of Ophthalmology, IRCCS-Fondazione Bietti, 00184 Rome, Italy; 5 Departmental Faculty of Medicine, UniCamillus-Saint Camillus International University of Health Sciences, 00181 Rome, Italy

**Keywords:** gabapentinoids, gabapentin, pregabalin, pharmacovigilance, eye disorders

## Abstract

**Background**: Gabapentinoids (gabapentin and pregabalin) are medications used to treat epilepsy and diabetic neuropathy. Reports of gabapentinoid-associated adverse drug reactions (ADRs) are increasing. This study aimed to explore these potential signals, with a focus on ocular adverse effects. **Methods**: A mixed-methods design was adopted, utilising quantitative and qualitative approaches encompassing: (I) a social listening approach using Reddit; (II) a pharmacovigilance retrospective disproportionality analysis study using serious reports to the FDA Adverse Event Reporting System (FAERS) (2015–2025); and (III) a literature review (2014–2025). **Results**: Reddit data revealed several user-reported adverse ocular effects, including blurred vision, diplopia and photosensitivity, across 78 and 40 identified reactions for gabapentin and pregabalin, respectively. Pharmacovigilance findings identified positive signals for eye disorders with both gabapentinoids compared to active control diazepam, amitriptyline and carbamazepine). Incidental findings highlighted potential discrepancies between FAERS data and undesired effects listed within the manufacturer’s literature, with pregabalin exhibiting higher reporting odds than gabapentin for cataract (ROR: 4.55; 95% CI: 2.51–8.26) and blindness (ROR: 2.63; 95% CI: 1.85–3.73). The literature review reinforced eye disorder concerns, specifically retinal effects with gabapentinoids, noting the absence of robust, high-quality research. Nevertheless, it identified a plausible biological mechanism involving the CACNA2D1 receptor in retinal cells of animal models, with evidence of an effect following oral administration of pregabalin. **Conclusions**: This study suggests an increase in eye disorder reports associated with gabapentinoid use. Further clinical and epidemiological studies are warranted to validate these real-world signals.

## 1. Introduction

Gabapentinoids are a class of drugs that includes pregabalin and gabapentin. European marketing authorisation encompasses seizures, peripheral neuropathic pain and anxiety only for pregabalin, with clinical use for other indications being off-label. Indications for gabapentin also include seizures and neuropathic pain, as well as other conditions including symptom management in multiple sclerosis. Gabapentinoids are believed to elicit their effects through binding to the α2δ-1 protein. Interaction between this protein and voltage-gated calcium channels, α-amino-3-hydroxy-5-methyl-4-isoxazolepropionic acid (AMPA) receptors and N-methyl-D-aspartate (NMDA) receptors, has implications for their synaptic trafficking and functioning [1], whilst upregulation of the protein has been associated with neuropathic pain processing [2]. Binding of gabapentinoids to the α2δ-1 protein may reduce neuropathic pain and neuronal excitability through disruption of the NMDA receptor complex, resulting in reduced trafficking and NMDA receptor-mediated excitation [1]. Inhibition of inflammatory mediators and potentiation of descending serotonergic pathways may also contribute to analgesic effects [3]. 

Due to the action of gabapentinoids on the central nervous system (CNS), expected side effects including memory loss, nausea and headaches arise [4,5]. However, emerging literature suggests serious physical and psychiatric adverse effects are under-researched. Since coming to the market in 1993 and 2004, respectively, gabapentin and pregabalin have been linked to several adverse drug reactions (ADRs) that have been explored through pharmacovigilance work. Fuzier et al. [6] investigated gabapentinoid ADRs reported to the French Pharmacovigilance System between 1995 and 2009, identifying lesser-known ADRs including hepatitis and haematological conditions.

Evidence of gabapentinoid misuse has been identified and characterised through European Medicines Agency EudraVigilance [7] and Food and Drug Administration (FDA) pharmacovigilance data [8]. Subsequently, in October 2018, a risk assessment was undertaken by the Advisory Council on the Misuse of Drugs (ACMD), which led to the reclassification of gabapentinoids to Schedule 3 controlled drugs in the UK [9], followed more recently by the introduction of stronger warning labels in prescribing information [10]. The ongoing concern regarding gabapentinoid misuse highlights the high-risk nature of these medications, warranting expansive research into all aspects of the medications, including adverse reactions.

Ongoing data review is crucial to support patient safety by ensuring prescribers have access to accurate information to allow them to make evidence-based decisions for their patients. Reports of ocular toxicity associated with gabapentinoid use have been occasionally reported in the literature [11,12,13], but to the best of our knowledge, have not been widely reviewed. Due to the ongoing investigation into the safety of gabapentinoids with regard to ADRs and misuse, this identified knowledge gap concerning ocular toxicity should be addressed to ensure the continued characterisation of these medicines’ safety profile.

This study aims to address a key knowledge gap by exploring potential under-reported ocular adverse effects via social media anecdotal evidence, pharmacovigilance analysis, and literature review, providing a foundation for future research into biological causation.

## 2. Results

### 2.1. Summary of Results Related to the Social Listening Study

**Figure 1:**
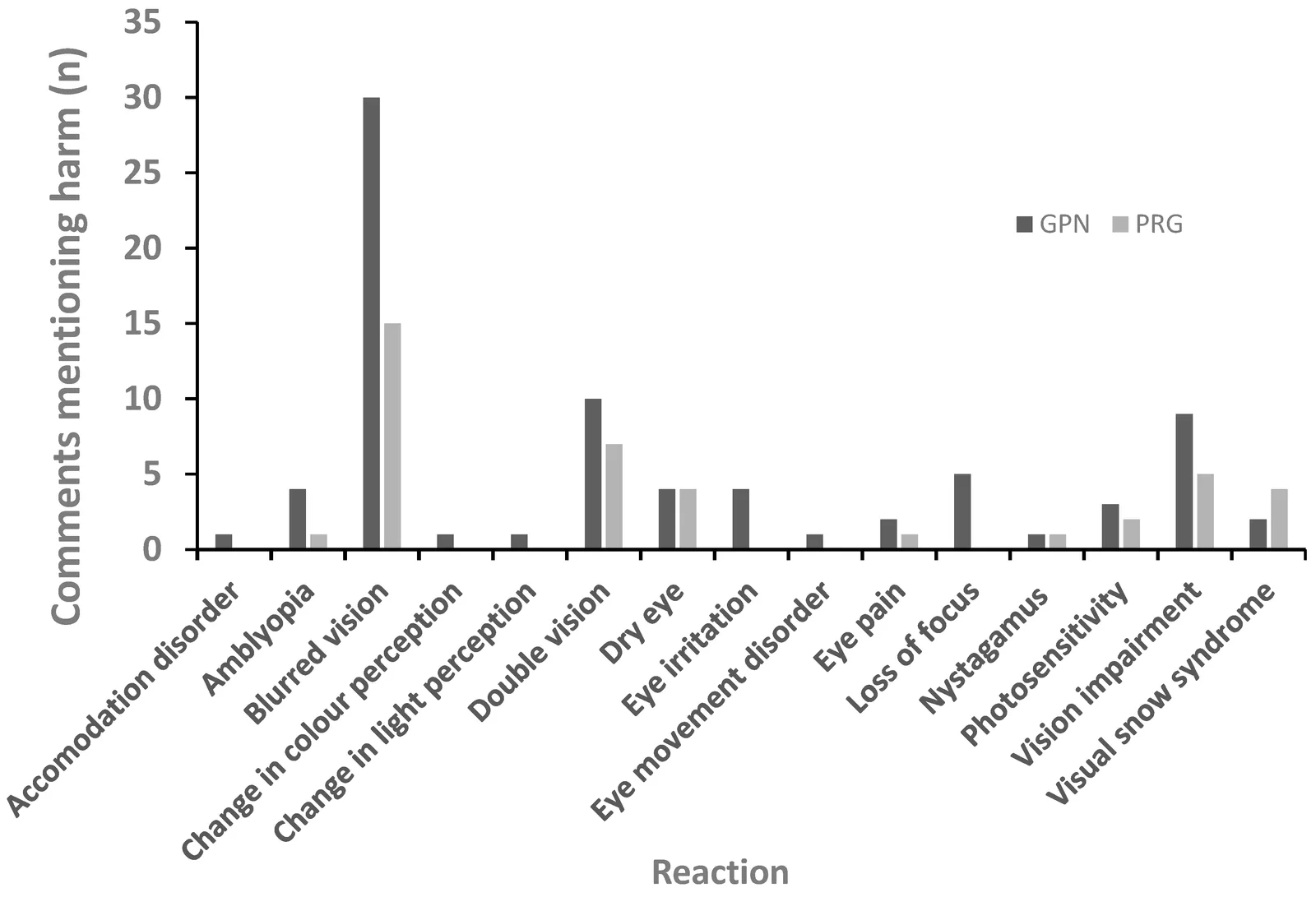
Reaction types and frequency reported by Reddit users collected during the social listening study. GPN: gabapentin; PRG: pregabalin.

A total of 84 Reddit comments mentioning gabapentinoids and vision disorders were identified, with estimated publication dates ranging from 2019 to 2025. Of these, 48 were related to gabapentin and 36 to pregabalin. A summary of the reaction type(s) for each comment and drug is presented in Figure 1. Blurred vision was the most commonly detected related harm for both gabapentin and pregabalin, followed by diplopia and vision impairment. More reactions were identified with reported use of gabapentin, with change in colour and light perception, loss of focus and accommodation disorder only appearing in the gabapentin search. In contrast, only visual snow syndrome was identified more with reported use of pregabalin than gabapentin. Dry eye was identified equally between reported use of each gabapentinoid.

### 2.2. Summary of Results Related to the Pharmacovigilance Study

Considering all reactions, 90,064 case listings were extracted for pregabalin; of these, 37,116 met the unmasked criteria and 12,890 met the unmasked serious criteria. A total of 67,426 case listings were extracted for gabapentin; of these, 21,458 met the unmasked criteria and 6294 met the unmasked serious criteria. A total of 33,269; 21,894; and 17,650 cases were extracted for diazepam, carbamazepine, and amitriptyline, respectively, with 2763, 5110 and 1207 cases meeting unmasked total criteria and 1421, 4099 and 15,689 cases meeting unmasked serious criteria, respectively. Unmasked, serious cases were the focus, and the aim was to hone findings to cases where the outcome was considered to constitute serious harm. To be defined as serious, the reaction resulted in death, was life-threatening, required hospitalisation or prolongation of existing hospitalisation, resulted in persistent or significant disability or incapacity, or constituted a birth defect [14]. Unmasked, total data are available upon request but have been omitted from this report to avoid duplication of signals.

Table 1 presents the retrospective disproportionality analysis results. More signals were identified in relation to pregabalin than gabapentin, with consistent findings across controls for blindness, cataract and eye disorder. Strong signals were identified in the comparison of pregabalin to gabapentin for eye haemorrhage, glaucoma and mydriasis, [15]; however, insignificant results were found in comparison to controls. Gabapentin results present signals associated with eye movement disorder and periorbital oedema, with some supportive data from controls for the former. Overall, the pharmacovigilance study elicited greater signals of significance for pregabalin than gabapentin.

Several ROR estimates exceeded 2.5 but did not reach statistical significance because the corresponding CIs were <1; these findings were therefore not considered statistically significant in this analysis.

### 2.3. Summary of Results Related to the Literature Review

The literature review identified 18 publications relating to ocular effects associated with gabapentinoids. The table below outlines the core aspects of each paper and the relevant findings (Table 2). Four studies were identified in relation to gabapentin, and 14 to pregabalin, emphasising the concentration of current research. Of these, six were case reports, four were pre-clinical studies and two were literature/systematic reviews, among various other cohort, observational and statistical studies. These studies range in their strength of evidence and focus area, ranging from patient experience reports to pre-clinical studies investigating potential mechanisms of action of gabapentinoids upon ocular structures. Therefore, more robust evidence that can be generated from clinical trials is needed to confirm the findings of this study.

## 3. Discussion

This study aimed to explore ocular adverse effects associated with gabapentinoids using a mixed-methods approach. To our knowledge, this is the first study to implement a mixed-methods design comprising a social listening study, a pharmacovigilance retrospective disproportionality analysis and a literature review to investigate potential ocular effects of gabapentinoids. The social listening study highlighted higher reporting of eye disorders associated with gabapentin than pregabalin, particularly blurred vision, double vision (diplopia) and vision impairment. Retrospective pharmacovigilance analysis signalled a wider variety of eye disorders related to pregabalin than those currently considered, and identified several signals related to gabapentin, suggesting a mechanistic effect involving ocular pathways. These signals are reported in accordance with MedDRA, whereby reactions are categorised by group, e.g., “Eye Disorder,” then further subcategorised by reaction by MedDRA preferred terms to enhance continuity of language. The literature review gathered a variety of research types, ranging from case studies reporting adverse ocular events linked to the use of gabapentinoids to pre-clinical studies supporting the consideration of mechanistic effects, identifying potential action of pregabalin on the CACNA2D1 receptor within the ocular pathways of a non-human species [21]. When considering this potential mechanism, it is necessary to consider other factors, including genetic vulnerability, vascular damage through diabetes and similar conditions, and age. These factors are likely confounding variables which, within the present study, limit the ability to suggest any further plausible biological mechanism from gathered data. Considering the pharmacovigilance study, utilising a wider range of sources and stratified input data, despite its inherent limitations, in conjunction with the literature review and current Summary of Product Characteristics (SmPC), it is evident that eye disorders, considered here, are more associated with pregabalin than gabapentin. It is also important to note that pregabalin is typically considered 5-6 times more potent than gabapentin; therefore, the consideration of an increasing effect depending on dose should not be disregarded.

The social listening study was congruent with the pregabalin SmPC’s listed adverse effects with regard to diplopia and blurred vision as common effects; here, these were the most reported side effects for pregabalin. Uncommon effects were also consistent, with reference to visual snow syndrome (visual field defects) and dry eye. However, in comparison to gabapentin, it was somewhat surprising that we gathered fewer comments related to pregabalin than gabapentin, given the higher emphasis on pregabalin eye disorders within the literature and SmPC [4,5]. Although the social listening data provided interesting anecdotal data, they cannot prove cause and effect between the use of gabapentinoids and eye disorders.

Within the pharmacovigilance analysis, pregabalin elicited a broader variety of positive signals relating to eye disorders, although most signals were identified in comparison to gabapentin. For serious cases (Table 1), eye haemorrhage, macular degeneration and mydriasis were more frequently reported in connection with pregabalin than gabapentin but failed to elicit similar results compared to controls. This was largely justifiable by the lack of comparative data available for eye haemorrhage and macular degeneration, suggesting these ADRs may be associated with pregabalin use only, although further research is required to rule out other potential causes. Other ADRs of note include blindness, blindness (unilateral), cataract and glaucoma, all of which provided statistically significant signals across two or more controls and gabapentin. This provides a stronger evidence base for the consideration of pregabalin linked to ocular adverse events, supported by the literature review. Several case reports listed pregabalin as a probable cause of various eye disorders, including serous macular detachment, photophobia and central serious chorioretinopathy [13,22,23]. Although inconsistent across the specific types of eye disorders, it may reinforce a clinical basis for a potential ocular biological effect, although robust research would be required. Updates have recently been made to the pregabalin SmPC to include “vision loss” and “keratitis,” [5] both of which have been identified in pharmacovigilance studies; however, discrepancies remain between the signals identified here, and within research. Macular effects have shown a positive signal and are highlighted within research; however, they have been omitted from the relevant SmPCs.

Although highlighting fewer signals compared to pregabalin, gabapentin elicited more signals than expected, considering the limited inclusion of eye disorders within the SmPC. Blurred vision was most reported by Reddit users which, while not listed within SmPC, may be misrepresented as a visual disturbance by members of the public due to its non-specificity as a symptom. Vision impairment was frequently reported—almost twice as much as pregabalin—however, it was not identified within the pharmacovigilance study; potentially, again, due to its non-specificity. However, it may be a symptom of a diagnosable condition, requiring ophthalmologic review. Overall, gabapentin was more prevalent in the social listening study, indicating that Reddit users may be more likely to discuss eye disorders related to gabapentin than pregabalin on this platform. The pharmacovigilance study identified signals for eye movement disorder and periorbital oedema; however, the later was only identified in comparison to pregabalin due to a lack of comparative data. Eye movement disorder showed strong signals across total and serious cases compared to pregabalin and statistically significant signals in comparison to carbamazepine but no other controls. These reactions differ greatly to those listed within SmPC due to the involvement of structural changes and muscle or nerve pathways rather than visual acuity itself. Due to the lack of comparable data, these signals should be verified using a larger database and if they are validated, further research into mechanism of harm should be considered. Case reports were identified within the literature (Table 2), particularly relating to macular effects, and the pharmacovigilance study also highlighted ocular neuromuscular disorders [12,27]. However, fewer large-scale studies were identified, suggesting a gap within the current evidence base relating gabapentin to eye disorders. Of the larger pre-clinical studies identified here, all were animal toxicology studies [26,30]. These studies identified pregabalin as potentially having an intraocular pressure-lowering effect, demonstrating an effect within the eye and adding to the evidence base to warrant further research in this area. A slightly different effect was realised with gabapentin, with researchers focusing on neuroprotective and oxidative-lowering effects [16]. Considering the similarity of gabapentin and pregabalin, it may be crucial to investigate these potential signals and ensure the risk of harm is not applicable to gabapentin.

## 4. Methods

A mixed-method approach was adopted, encompassing social listening and pharmacovigilance retrospective disproportionality analysis studies, and a literature review. This novel approach allowed anecdotal posts from social media users to inform the exploration of pharmacovigilance data, then subsequently combine findings with a literature review to further identify associations with under-reported ADRs putatively related to eye disorders. To our knowledge, this approach has not been previously explored in relation to gabapentinoids.

### 4.1. Social Listening Study

Reddit is a forum-based platform, whereby users join communities known as “SubReddits” and share content about shared interests [32]. The sample of analysed posts/comments was extracted from Reddit on 08/04/2025 by at 14:45 and 15:17 GMT for gabapentin and pregabalin, respectively, by MM. Search terms (“Gabapentin vision,” “Pregabalin vision”) were employed with no date limits. “Vision” was used in place of “eye” or similar to target adverse reactions and reduce irrelevant results. Additional eye-related terms were not employed because preliminary scoping indicated that broader descriptors (e.g., “eye”) substantially increased irrelevant results, whereas the use of “vision” provided greater specificity for identifying discussions of potential adverse events. Furthermore, Reddit discussions frequently use informal, non-scientific terminology, and increasing the number of specific search terms, including “preferred terms” (e.g., “blurred vision,” “ocular symptoms”) can reduce sensitivity by narrowing retrieval and potentially missing relevant posts. All identified posts and comments were then sorted by relevancy, determined by the Reddit site, and downloaded as PDFs. Relevancy was selected as the most appropriate sorting filter compared to alternatives, “Hot”, “Top”, “New” and “Comment count”, as it provided the most results applicable to the search criteria. Other filters were considered; however irrelevant results then appeared. The choice of filter may result in some selection bias; however, this was considered an acceptable risk to gather the most accurate data. Any duplicate posts or posts written by a bot, determined by the declaration, were disregarded; however, as no username information was collected, similar comments from the same user may have been recorded across different Subreddits. This is to protect user confidentiality and comply with Reddit user guidelines. All returned Subreddits were assessed for relevancy by title using inclusion/exclusion criteria as shown in Table 3. Relevant Subreddits and comments were then extracted into a Microsoft Excel (version no. 2505, 2025) spreadsheet. The details recorded here were date published, comment, summary of reported ADR [15], additional information such as indication and concomitant products, and the Subreddit it was published to. Collected comments were then reviewed by AG to ensure compliance with inclusion/exclusion criteria and MedDRA terminology. Inter-rater reliability was not formally assessed; however, no discrepancies were identified at any stage of the data extraction and validation processes. Analysis of relevant ocular adverse effects was undertaken in line with MedDRA definitions [15].

### 4.2. Pharmacovigilance Study

The FDA (Food Drug Administration) Adverse Event Reporting System (FAERS) (link provided in data availability statement) was utilised for data collection. This database collects spontaneous reports of adverse effects from consumers, manufacturers and healthcare providers and lists vital details regarding each case, including concomitant products, the suspect active ingredient, the reaction experienced and demographic information. Reports to FAERS between 2015 and 2025 (inclusive) were extracted to Microsoft Excel (version no. 2505, 2025) using the export table function for data, last reviewed 25 September 2025. This was repeated for gabapentin, pregabalin and three control drugs, ensuring all forms of available products were included. The controls chosen were diazepam, indicated for anxiety; amitriptyline, indicated for neuropathic pain; and carbamazepine, indicated for seizures. These were chosen because their listed indications overlap with those of gabapentinoids, allowing comparison of harm rates with other similar pharmacological treatment options. Brand names, salts and prodrugs of controls were included in the searches (Supplementary Information S1).

To extract and analyse adverse event data, a custom script was developed in Python (version 3.11.9) to process the FAERS case listings provided as comma-separated value (.csv) files. For each drug of interest, the programme accessed the corresponding FAERS dataset and identified reports containing eye disorders, defined according to the “*Eye disorders*” System Organ Class and its constituent Preferred Terms as specified in the FAERS database and in accordance with MedDRA terminology (Supplementary Information S2).

The script iteratively queried the dataset for each predefined eye disorder term and quantified the number of associated cases under two analytical conditions: (i) masked analyses, in which concomitant products and additional suspect active ingredients were permitted; and (ii) unmasked analyses, restricted to reports listing only the drug of interest as the suspect active ingredient, with no concomitant products recorded. For each drug, the programme generated a structured .csv output file summarising each eye disorder term and the corresponding number of cases.

For each drug of interest, two output files were produced: one including all reported cases (serious and non-serious), and a second limited to serious cases only, as classified within the FAERS dataset. Both files contained masked and unmasked case counts. Use of Python enabled systematic, consistent classification of eye disorder reports across large datasets and facilitated reproducible extraction of case counts suitable for comparative analysis.

The script was peer reviewed, tested, and refined by MM to ensure analytical robustness and consistency. Manual spot checks were conducted against the original FAERS Excel case listings to verify the accuracy of automated case identification and classification. All code files may be shared upon reasonable request.

A bidirectional cross-comparison was conducted in which eye disorder reports associated with gabapentin were analysed in comparison with pregabalin, and, conversely, eye disorder reports associated with pregabalin were analysed in comparison with gabapentin. Reporting Odds Ratios (RORs) were calculated as per Equation (1) and confidence intervals (CIs) as per Equation (2) [33]. Statistical significance was determined with a ROR of >2.5 and a CI > 1. Significance was determined at ROR > 2.5 to encompass the strongest signals available for comparison with controls. Statistically significant eye disorders were then assessed with total gabapentinoids against each control, then each gabapentinoid against each control. Control data was collected using the same code, with eye disorders to be searched for being the preferred terms deemed as statistically significant following initial gabapentinoid cross-comparisons.

(1) $$ROR=\frac{\left(A/B\right)}{\left(C/D\right)}$$

Equation (1) used to calculate ROR where *A*–*D* are values as identified in Table 4.

(2) $$CI=exp(LN(OR)\pm 1.96\times \sqrt{\frac{1}{A}+\frac{1}{B}+\frac{1}{C}+\frac{1}{D})}$$

Equation (2) used to calculate 95% CI where *A*–*D* are values as identified in Table 4.

Masked analyses were excluded from the primary evaluation because these reports permit concomitant products and additional suspect active ingredients, increasing the likelihood of confounding beyond the scope of this study. Accordingly, only unmasked data were included in the main analyses. Masked data were retained for completeness and can be made available upon reasonable request.

### 4.3. Literature Review

**Figure 2:**
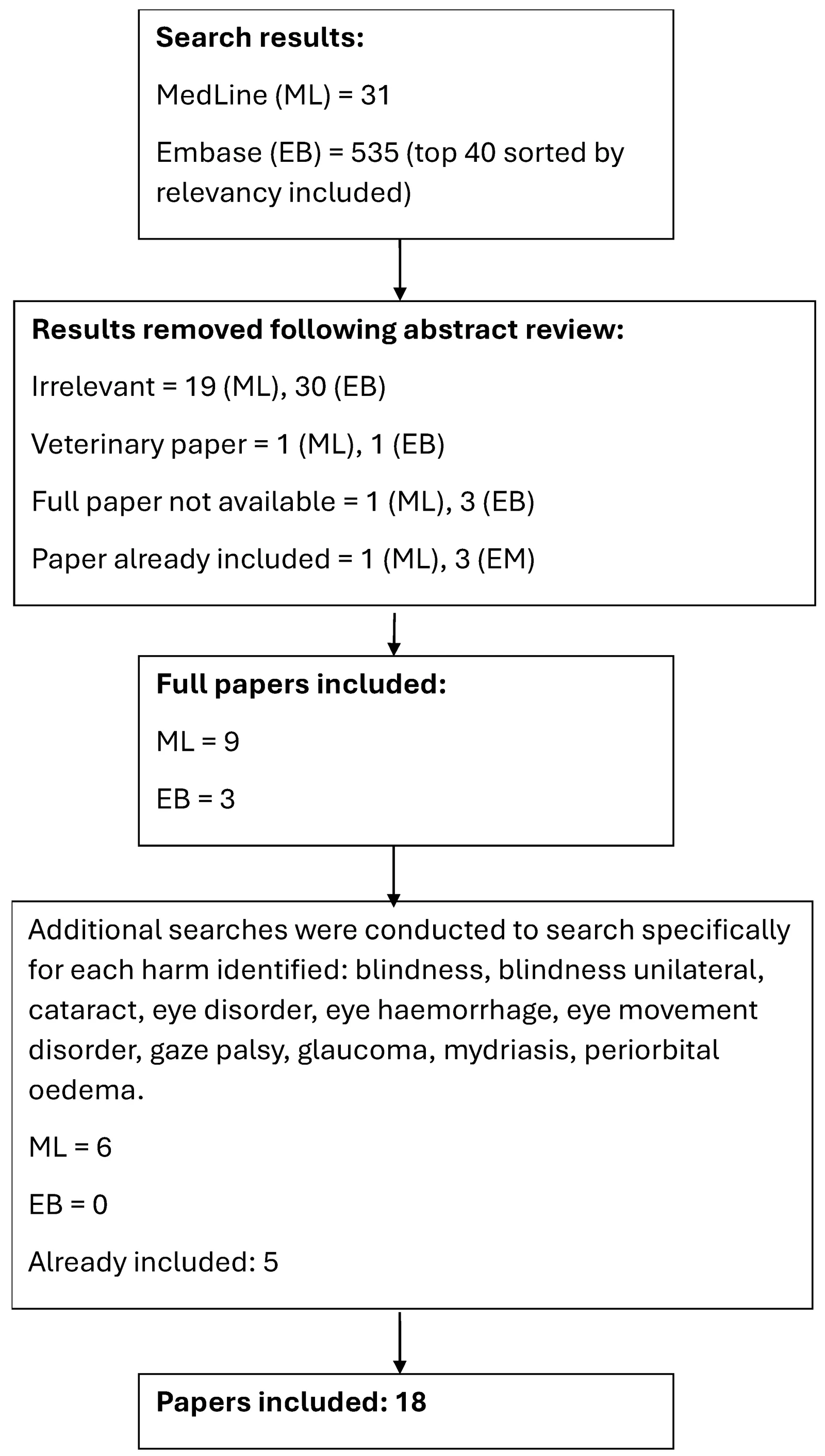
Flow chart summarising the exclusion process to collect relevant papers for the secondary literature review. Arrows indicate the flow of the process.

EBESCO host MedLine and Embase were utilised to conduct the literature search, for publication dates between January 2014 and February 2026. Search terms were placed into groups where Group one included gabapentinoids, pregabalin, gabapentin, and Group two included eye disorders, vision impairment and relevant synonyms. These terms were combined using Boolean operators (OR/AND) to produce a search string [34]. Inclusion/exclusion criteria, as follows, were applied. All formats of research and study types available were included, allowing for a broad selection of information and enabling thorough investigation from a variety of sources. Results not published in English or published before 2014 were excluded, ensuring only recent literature was identified and could be interpreted accurately, without dependency on online translation platforms. Abstracts were reviewed and assessed by the lead researcher and research supervisor for relevance; results deemed irrelevant to the search criteria were excluded (Figure 2).

Required information was extracted to Microsoft Excel (version no. 2505, 2025) to create a record of the: search term, DOI, title, authors, aim, geography, data used, outcomes, findings, data collection period and any additional information.

## 5. Conclusions

### 5.1. Strengths and Limitations

Although the social listening data gathered interesting anecdotal data, they cannot prove cause and effect between the use of gabapentinoids and eye disorders. Whilst this is a novel method of collecting user experience data, there is an inherent lack of clinical verification and self-reporting bias that must be considered when interpreting data. Comments from Reddit users could be under- or over-exaggerated, leading to discrepancies. To confirm a negative signal from the social listening study, additional researchers should be included to spot check interpretations, improving inter-rater reliability, or a Natural Language Processing model could be implemented for harm classification, improving reliability. Blindness is likely a rare harm; therefore, clinicians could be unlikely to attribute this to the medication, and patients may be under-educated on this possibility. Furthermore, blindness is a serious harm; therefore, users may not feel comfortable sharing this on a casual social platform, reducing the suitability of Reddit for this study. Information was not always available regarding concomitant products or other factors; therefore, causation links were also difficult to provide. A further potential confounding factor is the indication for which the gabapentinoid was being prescribed. Gabapentinoids are indicated for a range of conditions, including neuropathic pain. If the patient’s neuropathic pain were associated with diabetes mellitus, it is conceivable that any visual effects were a result of the patient’s condition, rather than the gabapentinoid.

The FAERS database is associated with widely understood inherent limitations due to the type of data collected. Ophthalmological-specific eye disorders such as mydriasis and cataract are reported within these results; however, details surrounding specialist investigations to be confident in diagnoses are omitted from collected data. Additionally, some discrepancies may arise between MedDRA definitions and FAERS reports. For example, eye haemorrhage is a general term encompassing intraocular haemorrhage, a broken blood vessel in the eye, eye bleeding, ocular haemorrhage and eye haemorrhage, although from an ophthalmological perspective this may mean subconjunctival haemorrhage. Therefore, caution is strongly encouraged when interpreting these terms and reference to MedDRA terminology is advised. In addition, inherent limitations relate to potential discrepancies in the definition of particular eye disorders reported anecdotally on Reddit versus their appropriate Eye Disorder MedDRA preferred terms used in reports on the FAERS database.

Furthermore, geographical bias may impact results, as 67.45% gabapentin and 62.06% pregabalin cases reported the United States as the country where the event occurred. This is expected, since the FAERS database is the national ADR reporting system for the US. This may not give an accurate representation of global prescribing trends and ADR reporting. Chan et al. [35] identified North America, Oceania and Northern Europe as having the highest prescribing of gabapentin in 2018 and collected FAERS reports originating from Great Britain made up only 6.7% of reports; therefore, it could be concluded that an international database would provide globally representative signals of eye disorders with gabapentinoid use. When comparing reporting rates between gabapentinoids and control drugs (amitriptyline, diazepam and carbamazepine), it should be noted that the incidence of reported ADRs may not be proportional to the number of items of each drug prescribed. Therefore, any apparent increased risk of an ADR may be a consequence of higher levels of prescribing of that drug.

Over- and under-reporting of ADRs, through pharmacovigilance reporting systems, is also a necessary consideration, given the voluntary nature of the submission process, potentially leading to multiple reports related to one case, skewing results, and the tendency for more serious reactions to be reported [36]. Additionally, confounding variables must be considered, such as the indication of gabapentin for diabetic neuropathy, [37] with diabetes being a predisposing risk factors for glaucoma and cataracts, among others. Given the lack of consistent “Reason for Use” data within FAERS, we cannot determine the impact of this upon signals identified, despite our best efforts to present only unmasked analysis, omitting any cases with concomitant products reported. Further detailed cohort studies with specific ophthalmology monitoring and medical history collection would be optimal to confirm or dispute the signals identified. The pharmacovigilance methods employed in this study used ROR > 2.5 to determine statistical significance, rather than the standard ROR > 1, to include the maximum amount of data whilst focusing on the strongest signals detected. Similar studies have determined this at ROR > 4 [38]; however, this was considered inappropriate for these data, as doing so would eliminate a large amount of control data, limiting discussion of signals. This should be considered in clinical interpretations.

Exclusion criteria applied within the literature review removed results not published in English. This may have removed results relevant to the study, including different trends within differing healthcare systems globally. However, provided translations were included, and results included reports from Turkey and Netherlands, demonstrating consistent findings throughout Europe. Additionally, we recognise the lack of directly relevant high-quality papers included within the literature review, with many being case reports, which naturally lack generalisation and may be susceptible to bias. These case reports do, however, include significant ophthalmic diagnostic details and therefore, due to the absence of current higher-level research, are appropriate to include and aim to increase the evidence base for such ADRs.

### 5.2. Recommendations

Identification of eye disorders as under-reported ADRs successfully addresses the aim, providing a strong basis for further research. Despite limited reporting within the social listening study, the pharmacovigilance provided consistent positive signals. The discrepancies within analysed FAERS data and current SmPC guidance warrant further investigations to ensure appropriate prescribing, particularly in patients with risk factors for eye disorders such as glaucoma. The literature review failed to identify any current robust research into eye disorders related to gabapentinoids; however, it did suggest a link between CACNA2D1 activation within the eye as a possible mechanism underpinning visual disturbance.

While potential risk of eye disorders may be small, it is important that patients and healthcare professionals are alert to potential ADRs. We recommend that any eye disorders potentially linked to medication use should be reported to relevant systems such as FAERS in the US, or the MHRA Yellow Card Scheme in the UK.

Further research into eye disorders attributed to short- and long-term gabapentinoid use should be conducted using a larger reporting database or a cohort study to investigate a larger sample and confirm findings. Regarding a biological causation for blindness associated with gabapentinoids, future research should focus on already identified receptors within animal toxicology studies, and their activity following gabapentinoid administration in short- and long-term patients.

## Figures and Tables

**Table 1:** Summary of unmasked, serious RORs for each comparison analysed for each reaction studied.

Reaction	GPN vs. PRG	GPN vs. DZP	GPN vs. AMT	GPN vs. CMP	PRG vs. GPN	PRG vs. DZP	PRG vs. AMT	PRG vs. CMP
Blindness	N/A	1.395 (0.59–3.31)	N/C	1.148 (0.67–1.97)	2.625 (1.85–3.73)	3.660 (1.62–8.26)	N/C	3.014 (1.92–4.73)
Blindness (Unilateral)	N/A	0.677 (0.07–6.52)	N/C	1.954 (0.20–18.79)	3.422 (1.02–11.48)	2.317 (0.31–17.24)	N/C	6.687 (0.90–49.73)
Cataract	N/A	1.355 (0.30–6.06)	N/C	0.977 (0.40–2.39)	4.547 (2.51–8.26)	6.163 (1.52–24.98)	N/C	4.442 (2.17–9.11)
Eye Disorder	N/A	0.338 (0.12–0.95)	N/C	1.173 (0.39–3.50)	2.610 (1.28–5.32)	0.881 (0.38–2.06)	N/C	3.060 (1.22–7.69)
Eye Haemorrhage	N/A	N/C	N/C	N/C	11.249 (1.51–83.32)	N/C	N/C	N/C
Eye-Movement Disorder	8.210 (2.74–24.57)	N/C	1.832 (0.24–13.84)	3.480 (1.01–11.95)	N/A	N/C	0.223 (0.03–2.00)	0.424 (0.10–1.90)
Gaze Palsy	N/C	N/C	N/C	2.826 (0.80–9.92)	N/A	N/C	N/C	0.000
Glaucoma	N/A	1.355 (0.16–11.26)	0.136 (0.04–0.45)	0.558 (0.19–1.66)	2.935 (1.23–6.97)	3.977 (0.55–29.03)	0.400 (0.16–1.02)	1.637 (0.73–3.68)
Mydriasis	N/A	0.020 (0.001–0.16)	0.008 (0.001–0.06)	0.065 (0.01–0.51)	9.779 (1.31–72.89)	0.199 (0.10–0.42)	0.078 (0.04–0.16)	0.635 (0.30–1.36)
Periorbital Oedema	7.175 (1.49–34.55)	N/C	N/C	N/C	N/A	N/C	N/C	N/C

Dark grey cells indicate ROR >2.5 and lower 95% CI >1. GPN (gabapentin), PRG (pregabalin), DZP (diazepam), AMT (amitriptyline), CMP (carbamazepine). N/C indicates non-calculable data (0 cases for comparative drug). N/A indicates that data was not calculated as a positive signal with the inverse analysis.

**Table 2:** Summary of included literature presented in chronological order for each gabapentin and pregabalin.

Year	Type of Study	Aim	Location	Data	Outcome	Comments	Reference
**Gabapentin**							
2016	Case report	Present details regarding case involving gabapentinoid use and occurrence of ocular effects.	South Korea	A 71-year-old male taking gabapentin and steroid for 8d presented to the clinic with macular oedema. Intraocular pressure was 9 mmHg (right eye) and 11 mmHg (left eye). Moderate cataract was also identified. Fundus examination showed macular oedema and serous retinal detachment of macular in both eyes.	After four weeks of stopping gabapentin, visual acuity improved and marked improvement of macular oedema and serous detachment was identified.	N/A	[12]
2019	Pre-clinical study	Studied the possible neuroprotective effects of gabapentin on streptozotocin-induced diabetic rat retina.	Saudi Arabia	Gabapentin treatment significantly reduced the levels of malondialdehyde and reduced oxidative stress by increasing levels of glutathione and reactive oxygen species (antioxidant).	Authors acknowledge case reports of macular oedema with gabapentin and recommend continued monitoring and further clinical trials	Non-human species.	[16]
2020	Case report	Present details regarding case involving gabapentinoid use and occurrence of ocular effects.	UK	A female patient in her late 40s who presented with severe visual impairment and Stevens–Johnson syndrome after using gabapentin for neck pain 10 months ago. Symptoms included lid margin keratinisation and symblepharon.	Case study suggested gabapentin as a cause of Stevens–Johnson syndrome	Also appeared in visual impairment search	[17]
2021	Literature review	To review the effect of drugs on the eye, specifying the area in which they can have an adverse effect to inform prescribers and professionals on management.	Australia	Cases of gabapentin-related nystagmus, diplopia and visual field defects have been reported.	No specific outcome, presenting consolidated findings from literature review.	Minimal data on gabapentin specifically. No mention of pregabalin.	[18]
**Pregabalin**							
2014	Case report	Present details regarding case involving gabapentinoid use and occurrence of ocular effects.	UK	A 30 yr old female patient gradually increasing pregabalin dose to 150 mg twice daily over 6 months reported vertigo and difficulty with execution of left eye pursuit.	Symptoms resolved when pregabalin tapered and stopped after 2 months.	N/A	[19]
2018	Case report	Present details regarding case involving gabapentinoid use and occurrence of ocular effects.	Turkey	A female patient who had bilateral serous macular detachment after attempted suicide using pregabalin.	Ophthalmology diagnosed bilateral serous macular detachment following overdose; likely cause was pregabalin	N/A	[13]
2019	Observational study	To investigate gabapentinoid dosing and adverse effects in Pakistani patients with neuropathic pain.	Pakistan	Observational study of 320 patients treated for neuropathic pain between 2016 and 2018. A total of 252 patients were followed up, of whom 206 received pregabalin. Visual blurring was noted in 3 patients receiving pregabalin only.	Visual blurring was noted in patients taking pregabalin but not gabapentin in this sample.	Overall, 51% of the sample were being treated for diabetic neuropathy.	[20]
2019	Pre-clinical study	Studied the possible neuroprotective effects of pregabalin in a rat model of diabetic retinopathy.	Egypt	Pregabalin reduced expression of retinal inflammatory markers, glutamate and Nox, and reduced optic nerve fibrosis.	Findings support a retinal protective effect of pregabalin and a potential role in the management of retinopathy.	Non-human species.	[21]
2020	Case report	Present details regarding case involving gabapentinoid use and occurrence of ocular effects.	Japan	A 49-yr old female patient with photophobia and night blindness in her left eye after 10 months of pregabalin administration.	One month after discontinuing pregabalin, the patient reported a subjective improvement in the photophobia and night blindness.	Oral pregabalin administration 50 mg/day. Also appeared in photophobia search.	[22]
2021	Case report	Present details regarding case involving gabapentinoid use and occurrence of ocular effects.	Turkey	Two cases of central serous chorioretinopathy, one in a 69-year-old female and another in a 45-year-old male	Pregabalin could be the cause of central serous chorioretinopathy.	One patient was taking concomitant products.	[23]
2021	Cohort study	To explore long- and short-sightedness associated with a range of medication including gabapentinoids, using UK Biobank.	UK	Pain relievers including gabapentinoids increased hyperopic refraction (longsightedness). These associations remained statistically significant after addressing potential sources of confounding and regardless of the subjects’ comorbidities or current activity levels.	The mechanism behind these associations is not entirely clear. However, given the heterogeneity of opiates, nonsteroidal anti-inflammatory drugs, paracetamol, and gabapentin, it is unlikely a direct effect. It was deemed not causative.	N/A	[24]
2021	Cross-sectional study	To explore the effects of long-term pregabalin use on the choroid and retinal nerve fibre layer.	Turkey	Forty-one patients taking pregabalin long term for fibromyalgia were compared to 41 newly diagnosed untreated controls. Pregabalin had no effect on the choroid thickness but had a thinning effect on the retinal nerve fibre layer.	The authors suggest avoiding long-term pregabalin in patients with RNFL damage and regular ocular examination.	Also identified in diabetic retinopathy search.	[25]
2021	Pre-clinical study	In silico and in vivo (rabbit) study looking at pregabalin and other identified compounds to investigate CACNA2D1 targeting and effect on intraocular pressure.	N/A	Pregabalin showed intraocular pressure-lowering effects. Effects of other drugs tested suggest CACNA2D1 is a validated drug target for treating glaucoma and is worthy of further investigation for more potent compounds.	Findings suggest pregabalin may act upon the CACNA2D1 receptor which induces a reduction in intraocular pressure.	Non-human species.	[26]
2022	Pharmacovigilance study	To evaluate the association of anti-seizure drugs including gabapentin to the occurrence of eye disorders using the FAERS database 2015–2020.	USA data	Several positive signals linking gabapentin to ocular neuromuscular disorders, diplopia, vision blurred and altered visual depth perception. Strong positive signals for hypotony maculopathy and mild positive signals for iritis.	Gabapentin was associated with adverse effects of the iris, macula, and retina.	Also present in diplopia or double vision and eye disorders searches.	[27]
2023	Pharmacovigilance study	To identify signals of drug induced glaucoma from the Japanese adverse event reporting database 2004 to 2017.	Japan	Pregabalin was one of the drugs most associated with glaucoma (37 reports). Pregabalin, oral n= 36 unadjusted ROR = 13.5 (95% CI= 9.65–19.0).	Pregabalin has a link to glaucoma occurrence in Japanese reports.	This is consistent with a nested case–control study. No mention of gabapentin.	[28]
2023	Systematic review—Pre-clinical	To identify agents that demonstrated ocular hypotensive activity in different animal species.	N/A	With topical administration of 0.9% pregabalin drops, IOP was reduced between 21 and 7% over 8 h post dose in dog, cat, and rabbit species.	Pregabalin can reduce intraocular pressure.	Not original data; data based on non-human species.	[29]
2024	Pre-clinical study	Evaluate pregabalin microemulsion (ME) formulation as a once daily glaucoma therapy. Determine pregabalin absorption into the eye.	USA	Pregabalin ME reduced IOP in non-human species by approximately 40% over 6 months. Miniscule drug concentrations were detected in frontal lobe and peripheral organs such as lungs, heart, and spleen. No pregabalin was detected in plasma and other peripheral organs such as kidneys and liver.	Pregabalin ME effectively reduced IOP in non-human species by approximately 40% over 6 months.	Non-human species.	[30]
2025	Disproportionate retrospective analysis	Utilised FAERS to collect data on the drugs most associated with blurred vision.	USA database (global reports possible)	Pregabalin ROR (95% CI): 4.15 (3.93–4.37); PRR (95% CI) 4.13 (4.07–4.18) when compared to other medication. Of the 119 drugs identified, pregabalin had the second greatest number of relevant reports (N = 1436) between 1 January 2004, and 30 September 2024.	Highlights pregabalin’s side effect profile regarding blurred vision. Suggests a dose-dependent effect.	N/A	[31]

UK: United Kingdom; USA: United States of America; N/A: global or unavailable location.

**Table 3:** Summary of inclusion and exclusion criteria employed throughout the social listening study data collection stage.

Inclusion Criteria	Exclusion Criteria
- Posts/comments mentioning adverse ocular effects associated with gabapentinoid use - Available posts/comments within a Subreddit opened within a year to date - Firsthand reports of users experiencing adverse ocular effects - Posts/comments potentially relating to illicit gabapentinoid use	- Posts/comments not mentioning adverse ocular effects - Posts/comments that are unclear or require excessive interpretation - Posts/comments relating to use in pets/animals - Posts/comments referring to a third party’s experience, e.g., a family member - Posts/comments comparing use of a gabapentinoid to another medication - Posts/comments where the user explicitly states doubts whether the gabapentinoid is the cause

**Table 4:** Table summarising alphabetical representations within equations shown in Equations (1) and (2).

	Number of Reports with Event of Interest	Number of Reports Without Event of Interest
Drug of interest	a	b
Comparator drug	c	d

“a” is the number of reports associated with the drug of interest (e.g., gabapentinoids), “b” is the number of reports not mentioning the event of interest associated with the drug of interest (e.g., all reports minus a), “c” is the number of reports associated with the event of interest relating to the comparator drug (e.g., diazepam) and “d” is the number of reports for the comparator drug with no mention of the event of interest (e.g., all reports minus c).

## Data Availability

Data supporting the reported results can be requested from the corresponding author. All Reddit posts are available upon request.

## Supplementary Materials

The following supporting information can be downloaded at: https://www.mdpi.com/article/10.3390/ph19081175/s1, Supplementary Information S1: Drugs of interest (including control drugs), their corresponding brand names, and relevant salts and prodrugs used as search terms for data collection from the FDA Adverse Event Reporting System (FAERS). Supplementary Information S2: Eye disorders included in the pharmacovigilance study, listed alphabetically and identified according to the Medical Dictionary for Regulatory Activities (MedDRA) terminology used in the FDA Adverse Event Reporting System (FAERS).

## Abbreviations

The following abbreviations are used in this manuscript:

ADR	Adverse drug reaction.
AMPA	α-amino-3-hydroxy-5-methyl-4-isoxazolepropionic acid.
CI	Confidence Interval (95%).
CNS	Central Nervous System.
FAERS	FDA Averse Event Reporting System.
FDA	Food and Drug Administration.
MedDRA	Medical Dictionary for Regulatory Activities.
NMDA	N-methyl-D-aspartate.
ROR	Reporting Odds Ratio.
SmPC	Summary of Product Characteristics.
