# Supplementary material for: Possible Signals of Ocular Disorders Associated with Gabapentinoids: A Three-Arm Study
Source: Pharmaceuticals (Basel). 2026 Jul 27;19(8):1175. doi: 10.3390/ph19081175 (PMC13516212; doi:10.3390/ph19081175)
Supplement: Supplementary file 1 [file pharmaceuticals-19-01175-s001.zip › pharmaceuticals-4409196-supplementary.pdf]

Supplementary Information S1: Drugs of interest (including control drugs), their corresponding brand names, and relevant salts and prodrugs used as search terms for data collection from the FDA Adverse Event Reporting System (FAERS).

**Pregabalin**	**Gabapentin**	**Diazepam**	**Amitriptyline**	**Carbamazepine**
Pregabalin (generic)	Gabapentin (generic)	Diazepam (generic)	Amitriptyline (generic)	Carbamazepine (generic)
Pregabalin (brand name)	Gabapentin (brand name)	Valium	Amitriptyline hydrochloride (generic)	Tegretol
Lyrica	Gabapentin Enacarbil		Amitriptyline hydrochloride (brand name)	Tegretol XR
Lyrica CR	Neurontin		Elavil	

Supplementary Information S2: Eye disorders included in the pharmacovigilance study, listed alphabetically and identified according to the Medical Dictionary for Regulatory Activities (MedDRA) terminology used in the FDA Adverse Event Reporting System (FAERS).

Abnormal Sensation In Eye

Accommodation Disorder

Age-Related Macular Degeneration

Altered Visual Depth Perception

Amaurosis

Amaurosis Fugax

Amblyopia

Amblyopia Strabismic

Angle Closure Glaucoma

Aniseikonia

Anisocoria

Anisometropia

Arteriosclerotic Retinopathy

Astigmatism

Asthenopia

Binocular Eye Movement Disorder

Blepharitis

Blepharospasm

Blindness

Blindness Cortical

Blindness Transient

Blindness Unilateral

Borderline Glaucoma

Cataract

Cataract Diabetic

Cataract Nuclear

Cataract Subcapsular

Central Serous Chorioretinopathy

Central Vision Loss

Chalazion

Charles Bonnet Syndrome

Chloropsia

Choroidal Neovascularisation

Chorioretinopathy

Chromatopsia

Ciliary Muscle Spasm

Colour Blindness Acquired

Conjunctival Cyst

Conjunctival Deposit

Conjunctival Discolouration

Conjunctival Haemorrhage

Conjunctival Hyperaemia

Conjunctival Oedema

Conjunctival Pallor

Conjunctivitis Allergic

Conjunctivochalasis

Contact Lens Intolerance

Corneal Bleeding

Corneal Deposits

Corneal Disorder

Corneal Epithelium Defect

Corneal Erosion

Corneal Infiltrates

Corneal Lesion

Corneal Oedema

Corneal Opacity

Corneal Scar

Cyanopsia

Cystoid Macular Oedema

Dark Circles Under Eyes

Dacryostenosis Acquired

Detachment Of Retinal Pigment Epithelium

Diabetic Eye Disease

Diabetic Retinal Oedema

Diabetic Retinopathy

Diplopia

Disorder Of Globe

Disorder Of Orbit

Dry Age-Related Macular Degeneration

Dry Eye

Dyschromatopsia

Dysmetropsia

Eczema Eyelids

Epiretinal Membrane

Episcleritis

Erythema Of Eyelid

Erythropsia

Excessive Eye Blinking

Exophthalmos

Extraocular Muscle Disorder

Eye Allergy

Eye Colour Change

Eye Degenerative Disorder

Eye Discharge

Eye Disorder

Eye Haematoma

Eye Haemorrhage

Eye Infarction

Eye Inflammation

Eye Irritation

Eye Movement Disorder

Eye Oedema

Eye Pain

Eye Pruritus

Eye Swelling

Eye Symptom

Eye Ulcer

Eyelid Bleeding

Eyelid Cyst

Eyelid Disorder

Eyelid Exfoliation

Eyelid Function Disorder

Eyelid Irritation

Eyelid Margin Crusting

Eyelid Myoclonus

Eyelid Oedema

Eyelid Pain

Eyelid Ptosis

Eyelid Rash

Eyelid Retraction

Eyelid Sensory Disorder

Eyelid Skin Dryness

Eyelids Pruritus

Flat Anterior Chamber Of Eye

Foreign Body Sensation In Eyes

Gaze Palsy

Glare

Glaucoma

Halo Vision

Heterophoria

Hippus

Holmes-Adie Pupil

Hyperaesthesia Eye

Hypermetropia

Hypoaesthesia Eye

Hypotony Of Eye

Iridocyclitis

Iris Disorder

Iritis

Keratitis

Keratoconus

Keratopathy

Lacrimation Decreased

Lacrimation Disorder

Lacrimation Increased

Lacrimal Disorder

Lacrimal Gland Enlargement

Lens Dislocation

Lens Disorder

Lenticular Opacities

Lid Sulcus Deepened

Macular Degeneration

Macular Detachment

Macular Fibrosis

Macular Hole

Macular Oedema

Maculopathy

Meibomianitis

Metamorphopsia

Miosis

Mydriasis

Myopia

Myopic Chorioretinal Degeneration

Narrow Anterior Chamber Angle

Neovascular Age-Related Macular Degeneration

Night Blindness

Normal Tension Glaucoma

Ocular Discomfort

Ocular Hyperaemia

Ocular Hypertension

Ocular Sarcoidosis

Ocular Vascular Disorder

Oculogyric Crisis

Open Angle Glaucoma

Ophthalmoplegia

Optic Atrophy

Optic Disc Disorder

Optic Disc Haemorrhage

Optic Disc Vascular Disorder

Optic Ischaemic Neuropathy

Optic Nerve Cupping

Optic Nerve Disorder

Optic Nerve Infarction

Optic Nerve Sheath Haemorrhage

Optic Neuropathy

Orbital Myositis

Orbital Oedema

Oscillopsia

Papilloedema

Periorbital Disorder

Periorbital Oedema

Periorbital Pain

Periorbital Swelling

Photokeratitis

Photophobia

Photopsia

Posterior Capsule Opacification

Presbyopia

Proliferative Vitreoretinopathy

Pseudomyopia

Pseudophakia

Punctate Keratitis

Pupil Fixed

Pupillary Disorder

Pupillary Reflex Impaired

Refraction Disorder

Retinal Aneurysm

Retinal Artery Embolism

Retinal Artery Occlusion

Retinal Artery Thrombosis

Retinal Degeneration

Retinal Depigmentation

Retinal Deposits

Retinal Detachment

Retinal Disorder

Retinal Exudates

Retinal Haemorrhage

Retinal Infarction

Retinal Ischaemia

Retinal Oedema

Retinal Pigmentation

Retinal Scar

Retinal Tear

Retinal Toxicity

Retinal Vascular Disorder

Retinal Vascular Thrombosis

Retinal Vasculitis

Retinal Vein Occlusion

Retinal Vein Thrombosis

Retinopathy

Retinoschisis

Scleral Discolouration

Scintillating Scotoma

Scleritis

Serous Retinal Detachment

Strabismus

Subretinal Fluid

Sudden Visual Loss

Swelling Of Eyelid

Swollen Tear Duct

Tolosa-Hunt Syndrome

Trichiasis

Ulcerative Keratitis

Uveitis

Vision Blurred

Visual Acuity Reduced

Visual Acuity Reduced Transiently

Visual Brightness

Visual Field Defect

Visual Impairment

Vitreous Adhesions

Vitreous Degeneration

Vitreous Detachment

Vitreous Disorder

Vitreous Floaters

Vitreous Haemorrhage

Vitreous Haze

Vitreous Opacities

Xanthopsia

Xerophthalmia
